# A Global Index to Quantify Discrimination Resulting from COVID-19 Pandemic Response Policies

**DOI:** 10.3390/ijerph22040467

**Published:** 2025-03-21

**Authors:** Claus Rinner, Mariko Uda, Laurie Manwell

**Affiliations:** 1 Department of Geography and Environmental Studies, Toronto Metropolitan University, Toronto, ON M5B 2K3, Canada; 2 Faculty of Science, Wilfrid Laurier University, Waterloo, ON N2L 3C5, Canada

**Keywords:** COVID-19, discrimination, pandemic response, public health, vaccination status

## Abstract

Immediately following the emergency use authorizations of COVID-19 vaccines, governments around the world made these products available to their populations and later started implementing differential rules for vaccinated and unvaccinated citizens regarding mobility and access to venues and services. The Oxford COVID-19 Government Response Tracker (OxCGRT) is a time series database that reflects the extent of public health measures in each country. On the basis of the OxCGRT Containment and Health Index, we calculated a corresponding discrimination index by subtracting the daily index values for vaccinated and unvaccinated individuals. The resulting metric provides a cursory quantification of the discrimination experienced by unvaccinated individuals throughout 2021 and 2022. Patterns in the index data show a high degree of discrimination with great numeric and temporal differences between jurisdictions. Around 90% of countries in Europe and North and South America discriminated against their unvaccinated citizens at some point during the pandemic. The least amount of discrimination was found for countries in Central America and Africa. In order to move towards sustainable post-pandemic recovery and prevent discriminatory public health policies in the future, we recommend that human rights protections be expanded and the prohibition of discrimination be extended beyond a limited list of grounds.

## 1. Introduction

In response to the COVID-19 pandemic, governments around the globe have supported the expedited development of vaccines that were then promoted in population-level public health campaigns as safe and effective in reducing severe illness and death, as well as disease transmission [1,2,3]. COVID-19 vaccines were also promoted as a pandemic exit strategy, as the “only way out” of the crisis [4]. This coordinated global effort is perhaps best illustrated through the number of different vaccines being developed. According to the London School of Hygiene & Tropical Medicine’s COVID-19 vaccine tracker [5], there were 356 vaccine candidates, of which 138 entered clinical trials and 34 were in use in at least one jurisdiction by the site’s last update on 11 August 2022. The American “Operation Warp Speed” alone cost USD 18 billion [6].

These vaccines were generally offered for “free”, i.e., taxpayer-funded, as soon as emergency approvals were granted starting in late 2020 [7]. Healthcare workers were among the first groups who were expected or even required to be vaccinated [8]. By summer or fall 2021, increasing pressure was exerted on the general population of most countries to “get their shots” [9,10]. In addition to mass public health communications [11,12], vaccination mandates appeared in non-medical workplaces [7] and post-secondary institutions [13], and vaccination passports were created to restrict the mobility and social participation of unvaccinated citizens [7,14,15]. In conjunction with the presumed community benefits of vaccination against COVID-19, vaccination passes/passports were generally considered legal and legitimate, and only rare exemptions were granted on the basis of tightly defined medical and religious grounds [16,17].

With respect to the ethics of promoting and mandating COVID-19 vaccination, most researchers have been concerned with “vaccine equity”, e.g., [18,19,20,21]. Questions of distributive justice can take unexpected turns, as, e.g., some Canadians traveled to the US in order to gain access to the single-shot, non-mRNA Janssen (J&J) product that was not widely available in Canada. Researchers also studied the reasons behind the “anti-vax” sentiment of the remaining “hold-outs” [22,23], yet valid reasons for COVID-19 “vaccine hesitancy” were rarely considered [24]. Similarly, concerns about the impossibility of fully informed voluntary consent to COVID-19 vaccination, which were raised in conjunction with the trials [25], workplace mandates [26], mass vaccination campaigns [27], and vaccine passports [13], were never meaningfully addressed. The ethical principles underpinning the right to refuse vaccination, asserted by Kowalik [28] as a matter of innate bodily autonomy, also garnered little attention in academia or published opinion. Meanwhile, the lack of robust evidence for a predominant role of unvaccinated individuals in virus transmission [29,30], the superiority of vaccine-induced immunity over natural immunity [31], or the existence of a “pandemic of the unvaccinated” [32] should have led to more inclusive policies and attitudes. Instead, skewed risk perception and politics resulted in prejudice against the unvaccinated [33].

Possibly a result of mass public vaccine promotion campaigns [11], discriminatory attitudes against unvaccinated people increased and were found to be stronger than those held against some traditionally marginalized groups in the United States [34]. In a multi-layered cross-cultural study conducted in 21 countries with over 60,000 participants, Bor et al. [34] examined affective, cognitive, and attitudinal expressions of prejudice between groups with and without COVID-19 vaccination. The three concrete attitudes—antipathy, family exclusion, and restrictions of rights and freedoms—were unidirectional, targeting unvaccinated people. Other researchers have studied the “scapegoating of the unvaccinated” in English-language media [35]; global experiences of “unjustified discrimination” of the unvaccinated [36]; social sorting processes based on COVID-19 vaccination status resulting in discrimination and stigmatization [37]; and vaccination mandates as discriminatory measures against specific groups such as healthcare students in Ireland [38]. Wüstner [39,40] similarly deconstructs the political narratives around vaccine denial and their possible long-term social consequences. In yet another survey, Wüstner [41] confirmed that half of the respondents supported discrimination and punishment of unvaccinated German citizens.

The United Nations Universal Declaration of Human Rights stipulates that “Everyone is entitled to all the rights and freedoms set forth in this Declaration, without distinction of any kind, such as race, color, sex, language, religion, political or other opinion, national or social origin, property, birth or other status” [42], emphasis added by the authors]. In contrast to, e.g., Smith and Emanuel [43], we assert that discrimination is not conditional upon a predefined list of grounds. For example, the UN Declaration’s Article 7 on equal protection against discrimination does not list any limiting grounds at all. Based on the etymology of the word “discrimination”, from Latin to distinguish or discern, it is common sense that the application of differential policies to vaccinated and unvaccinated populations constitutes discrimination. The Wiktionary [44] defines discrimination as the “differential treatment of an individual or group to their disadvantage; treatment or consideration based on class or category rather than individual merit” with synonyms “partiality; prejudice; bigotry”.

On the basis of these observations and in the broader context of reassessing the COVID-19 pandemic response, we set out to quantify discrimination of “the unvaccinated” at a country level and examine its magnitude and spatio-temporal patterns. Our discrimination index reflects differential access to services, venues, and mobility for groups considered vaccinated or unvaccinated, respectively. The remainder of the manuscript outlines the data sources and methods of analysis, key findings along with conclusions and an outlook on further work.

## 2. Materials and Methods

The Oxford COVID-19 Government Response Tracker (OxCGRT) collected information on pandemic response measures enacted by governments around the world during 2020, 2021, and 2022, recording the extent of 24 measures, using 24 policy indicators, on a daily basis for 185 countries [45]. The policy indicators were grouped into four broad categories: containment and closure policies (C), economic policies (E), health system policies (H), and vaccination policies (V) [46]. Each policy indicator (e.g., school closing) is coded to describe the extent of the measure, as shown in Table 1 (e.g., for school closing: 0 = no measures; 1 = recommend closing or open with alterations; 2 = require closing, some; 3 = require closing, all). The dataset is complete since all indicators start at 0 and were only changed when the OxCGRT team found published evidence of a change in measures. From some time in 2021 onwards, ten of the C and H indicators differentiate between policies applicable to vaccinated and unvaccinated individuals, thus allowing for our quantitative analysis of discrimination.

The OxCGRT project also grouped some of the C, E, and H policy indicators in different ways to form four pre-calculated composite indices: Government Response Index, Containment and Health Index, Stringency Index, and Economic Support Index [46]. Table 1 shows the 14 indicators that constitute the Containment and Health Index (CHI). Note that the better-known Stringency Index is similar to the CHI, composed of the same C indicators, but includes only one H indicator, H1. The scores for the OxCGRT indices are calculated by taking each policy indicator code, subtracting 0.5 for regionally targeted measures (flag code = 0), normalizing the resulting value on a scale of 0 to 100, and then averaging over all indicators. Details on the calculation of this equally weighted composite index are explained in the file *documentation_and_coding.md* version 1, June 2023 [47] and in the Oxford project’s final working paper [46]. The reason given by the OxCGRT researchers for subtracting 0.5 for regionally targeted measures, is that the policy indicator codes are assigned based on the strictest policy found across subnational jurisdictions and thus may exaggerate overall country policy strengths. This subtraction applies a crude correction.

Indicators C1 to C8, H6, and H8 “reflected differentiated policies for vaccinated and non-vaccinated individuals” [46], providing both “vaccinated” and “non-vaccinated” values for periods when a differentiation was in effect. The definition of what constitutes “vaccinated” status (vaccine brands, number of doses) reflects each country’s policy stipulations and may thus differ between countries. These differentiated policies are, in general, referring to restrictions in place for the general public, not for employees. Thus, for policy indicator C1 ‘school closing’, the differentiated policies apply to students, not teachers; C2 ‘workplace closing’ targets customers; C3 ‘cancel public events’, attendees; C5 ‘close public transport’, passengers; and H8 ‘protection of elderly people’, care home residents and visitors [46] (p. 83).

**Figure 1:**
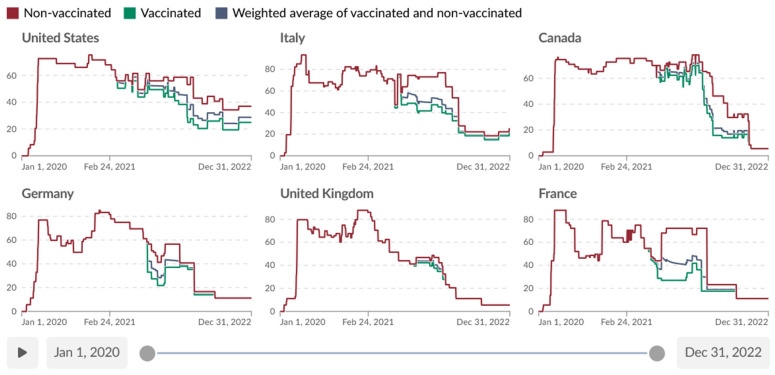
Daily values of the OxCGRT Stringency Index from 5 January 2020, to 31 December 2022, with differentiation of pandemic response policies applied to vaccinated and non-vaccinated individuals. Source: Screenshot of Our World in Data [49], licensed under Creative Commons CC-BY 4.0).

Given that indicators C1 to C8, H6, and H8 are differentiated, both the Containment and Health Index and Stringency Index are differentiated. Hale et al. [46] (pp. 24–29) provide an overview of “Differentiated policies based on vaccination status” as of 8 August 2022. This includes the timelines of the differential Stringency Index globally and for selected countries, as well as the global averages for non-vaccinated and vaccinated “policy strength” for selected individual indicators, without turning the difference into a metric of its own. Figure 1 from the Our World in Data (OWID) Coronavirus website [48,49] illustrates the 2020–2022 timeline of the Stringency Index for six selected countries, with the area between the red and green curves representing periods and magnitudes of discrimination of unvaccinated people.

The data for our analysis were retrieved from the archived version 1 of OxCGRT [47], current as of June 2023. Of the three years 2020–2022 that are available, only 2021 and 2022 were of interest due to the timing of the global rollout of COVID-19 vaccines. The two files *OxCGRT_fullwithnotes_national_2021_v1.csv* and *OxCGRT_fullwithnotes_national_2022_v1.csv* contain daily values for the 24 policy indicators and four index variables.

In order to be able to analyze the country data by continents, a list of 249 countries by continent was obtained from the StatisticsTimes.com web site at https://statisticstimes.com/geography/countries-by-continents.php (accessed on 13 September 2023). The original source specified on the site is the United Nations Statistics Division with a current date of 22 October 2019. This dataset included country names, 3-letter ISO codes, world region, and continent.

The OxCGRT data were reduced to only the CHI and stacked to combine the two (vaccinated and unvaccinated) annual time series for each country in a single dataset with a continuous time axis. We selected the CHI, which includes all ten differentiated indicators tabulated in the appendix of [46]. The daily index values are within a range of 0.0 to 100.0, with greater values representing tighter restrictions or further-reaching policies. The values for the vaccinated were then subtracted from the values for the unvaccinated, resulting in our discrimination index:

Discrimination_Index = CHI_NonVaccinated − CHI_Vaccinated

**Figure 2:**
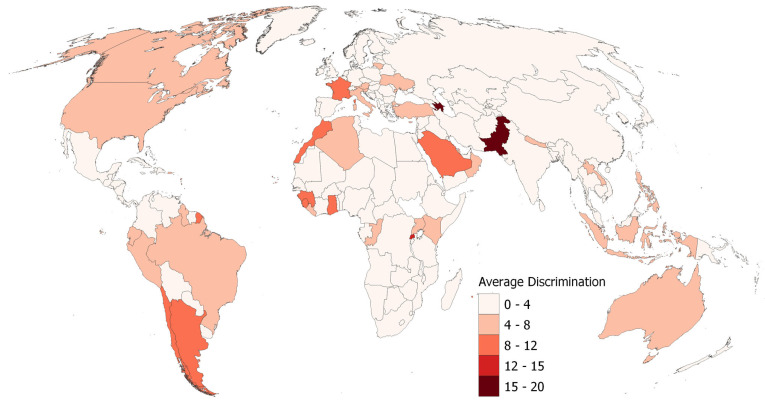
Average levels of discrimination in 2021–2022 by country calculated on the basis of OxCGRT’s differentiated Containment and Health Index. Data source for country boundaries: naturalearthdata.com (public domain, accessed on 29 September 2024).

The processed data were uploaded to an interactive Tableau Public visualization at https://public.tableau.com/app/profile/claus.rinner/viz/QuantifyingDiscrimination/Fig_2_Averagediscrimination (accessed on 15 March 2025). The data table was linked to the countries-by-continent list by matching Country Code with ISO-alpha3 Code. A horizontal bar chart was created for the average discrimination index value of each of the 185 countries, along with the maximum discrimination and the underlying average CHI. Testing for normality of the discrimination scores suggested adding the median discrimination index values as well. Due to longer phases of zero discrimination in the earlier phase, the bar chart shows that many countries had medians of zero and the average (mean) best represents the combination of duration and magnitude of discrimination over the entire study period.

The Tableau visualization also includes maps of the two-year average and two-year maximum discrimination index values per country. Five classes were used to symbolize equal intervals of growing discrimination values along an increasingly dark color ramp. For this article, we used QGIS to recreate these maps with proper cartographic projection (Mollweide). Lastly, a line chart for the discrimination index was created by plotting daily index values against the *date* variable, with *Country Name* representing the primary geographic dimension and colors associated with the six available continents. All charts in Tableau offer user interactions such as data display on mouse hover; selection and highlighting of individual curves, and sorting the country list by index values in ascending or descending order.

## 3. Results

### 3.1. Countries with Highest and Lowest Levels of Discrimination

Around 90% of countries in Europe and North and South America discriminated against their unvaccinated citizens in terms of access to services, venues, and mobility, while only two-thirds of African and Asian countries did. Less than one quarter (43 out of 185) of countries included in this study were not recorded for any discriminatory pandemic response measures according to OxCGRT’s Containment and Health Index. This group primarily includes African (18) and Asian (15) countries.

Based on the linear trendline in the Tableau scatterplots, higher average discrimination values were strongly associated with higher *maximum* discrimination values (R-squared = 0.78, *p* < 0.0001). Moreover, higher average discrimination values were also associated with higher values of the underlying CHI, albeit to a lesser degree (R-squared = 0.16, *p* < 0.0001). In other words, tighter restrictions entailed greater differentiation by vaccination status at a global scale.

Table 2 lists the countries with the highest levels of average or maximum discrimination based on the CHI. Five countries feature in both, the top 10 by average and by maximum: Azerbaijan, Pakistan, Rwanda, Cape Verde, and Saudi Arabia. This means that the countries have both reached a high maximum level of discrimination between unvaccinated and vaccinated citizens at one point in the 2021–2022 period, as well as sustained levels of discrimination throughout the period that were high enough to achieve a high average. Counter examples include France, Ecuador, Turkey, Kosovo, and Oman, which made the top 10 for maximum discrimination but not for average discrimination.

In France, although there was widespread public protest in the hundreds of thousands [50], vaccine passports for many areas of public access were instituted by the government, going as far as requiring a booster dose for those 65 years of age and older to remain valid [51]. Based on the notes accompanying the OxCGRT [47] data, France’s high level of maximum discrimination was most prevalent during the period of 16 February to 13 March 2022, when the vaccine passport continued to exclude unvaccinated people from many places of leisure and culture, trade fairs, exhibitions, seminars, etc., as well as inter-regional public transport, while restrictions were essentially eliminated for the vaccinated with the reopening of discotheques, nightclubs, and similar venues. Additionally, unvaccinated visitors from countries with active circulation of the virus (coded “orange”) continued to only be able to enter France if they had a compelling reason.

Other notable examples include Azerbaijan, Pakistan, and Rwanda. In Azerbaijan, early introduction of vaccine passports was enforced through arrests and fines for unvaccinated citizens who violated gathering restrictions and later the passports prevented them from accessing most public areas [52]. In Pakistan, the government imposed extensive restrictions including bans on unvaccinated citizens from entering public offices, schools, restaurants, public transport, retail establishments, and air travel, with enforcement through blocking cell phone SIM cards for people without required vaccination certificates [53]. In Rwanda, the government’s swift and authoritarian response to the pandemic has been described as “success and solidarity but at the price of democracy” [54]. Prevention measures included early and sweeping restrictions, such as lockdowns and curfews, enforced by armed police who even detained citizens believed to be in breach of restrictions in a stadium without any due process [54], and reports of forced vaccination by armed police in villages similar as occurred in other African countries [55,56], with some Rwandans seeking asylum from forced vaccination in the Democratic Republic of Congo [57]. Unvaccinated people in Rwanda faced severe discrimination and were denied access to all but essential businesses, gatherings (except funerals), and public transit for much of 2022, based on the notes accompanying the OxCGRT data.

The average CHI for countries with no recorded discriminatory policies is 39.16, compared with a much higher level of restrictions of 49.49 among the countries that did enact differential rules by vaccination status, The largest zero-discrimination countries by population are China, Ethiopia, Vietnam, Thailand, and Tanzania. China is known for its harsh lockdown measures and does indeed have the highest CHI values in the dataset with an average across 2021–2022 of 79.43. Interestingly, Zhu [58] presents the Chinese policies as a trade-off allowing the country to avoid vaccination mandates. Tanzania at the other extreme had lax government interventions with an average CHI value of only 17.75. In early 2021, there was little acknowledgement of COVID-19 deaths, little testing, and government officials promoted exercise, nutritious food, and herbal medicine, and steam inhalation over vaccines, which they were skeptical of [59], but later administered en masse [60]. The three other large zero-discrimination countries had average CHI values between those of China and Tanzania (Vietnam 60.30, Ethiopia 48.32, and Thailand 46.65).

### 3.2. Geographic Patterns of Discrimination

Among mid-sized and larger countries by land mass, which are discernible on the following maps, Pakistan and Azerbaijan stand out with the highest levels of discrimination close to 20 averaged over time (see Figure 2). Rwanda, the third country in the top 10 (see Table 2) is associated with the second value interval, ranging from 12 to 16 (very difficult to identify on the map). Next, France, Morocco, and Sierra Leone, along with Saudi Arabia as well as Argentina, Chile, and Fiji, follow in the middle class with average discrimination values around 8 to 12. That class also includes countries outside the top 10, including two additional West-African countries, Ghana and Guinea. Large swaths of North and South America, Southeast Asia, Australia, and a few southern and eastern European countries fall into the fourth class with values ranging from 4 to 8, while Central America, half of Europe, and much of Africa and Asia including Russia, China, and India, as well as New Zealand, had among the lowest average discrimination as measured by the containment and health metric.

**Figure 3:**
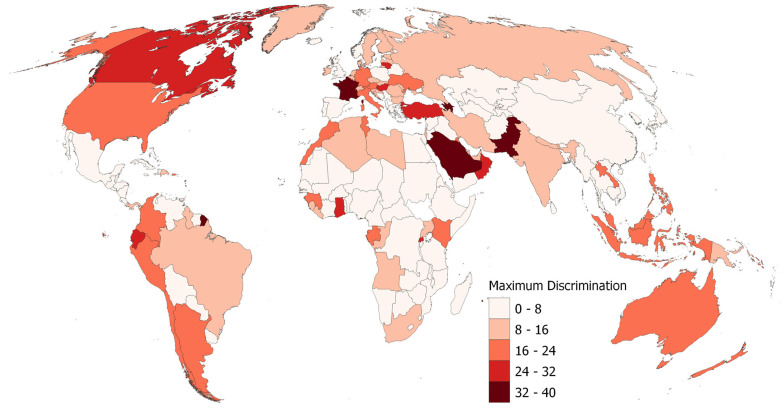
Maximum levels of discrimination in 2021–2022 by country calculated on the basis of OxCGRT’s differentiated Containment and Health Index. Data source for country boundaries: naturalearthdata.com (public domain, accessed on 29 September 2024).

A more varied spatial pattern, i.e., greater differences between neighboring countries, can be observed when looking at the maximal degree of discrimination reached (see Figure 3). For example, European countries span all five classes, defined by equal numeric intervals between 0 and 40. This includes France with one of the highest maxima as indicated in its top-10 position in Table 2; Hungary, Lithuania, and the top-10 country Turkey in the second highest grouping; Germany, Austria, and Italy in the middle; Ireland, Benelux, and a number of northern and eastern European countries in the second lowest grouping; and the UK, Spain, Portugal, Iceland, Norway, and a few southeastern European countries displaying the lowest levels of maximum discrimination. The maximum levels in North and South America were considerable overall, while Central America and much of Africa displayed low values. Similarly, northern and central Asian countries (with the notable exception of top-10-ranked Pakistan) tend to have low maxima, while Southeast Asia and Oceania display medium levels of maximum discrimination.

### 3.3. Timeline of Discrimination

**Figure 4:**
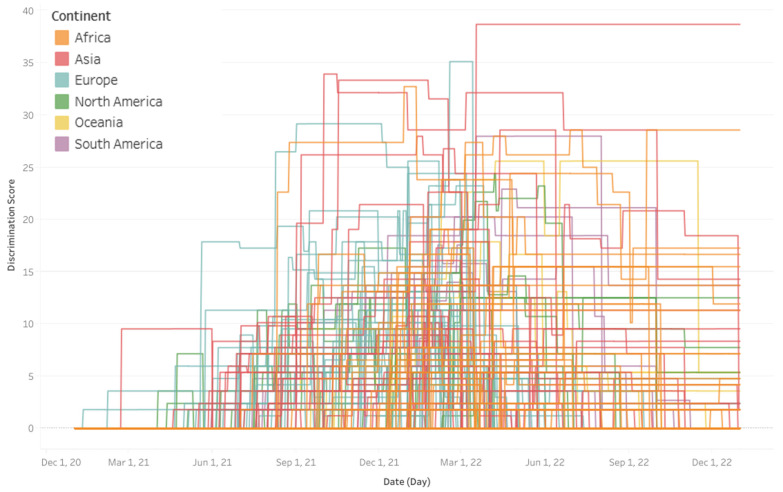
Timeline of discrimination levels in 2021–2022 by country calculated on the basis of OxCGRT’s differentiated Containment and Health Index.

When examining the timeline of discriminatory pandemic response policies (Figure 4), groups of early and late adopters, as well as timing and places of the greatest levels of discrimination, can be identified. Among the first countries to differentiate pandemic response policies by vaccination status are Estonia, Sweden, and Israel in January and February 2021, followed by the United States, Belize, Lebanon, Denmark, and Bahamas in April 2021. The bulk of discriminatory policies were enacted starting between May and September 2021, with some countries to follow as late as December 2021 (e.g., Rwanda) or January 2022 (e.g., Algeria).

High levels of discrimination over 25 were reached in August 2021 by France and Cape Verde, followed in September 2021 by Turkey. In October 2021, Azerbaijan was the first to exceed 30, joined later by Cape Verde (December 2021) and France (February 2022). By mid-March 2022, Pakistan had reached a discrimination value of close to 39. This value persisted until the end of 2022, making Pakistan the worst and longest-lasting hold-out in terms of discriminatory pandemic response. Rwanda displays another high and lasting value of close to 29 until year-end, and several other African countries maintained medium degrees of discrimination, including Ghana, Morocco, Sierra Leone and Liberia, as well as Indonesia and Azerbaijan in Asia, with values ranging from about 14 to 19.

**Figure 5:**
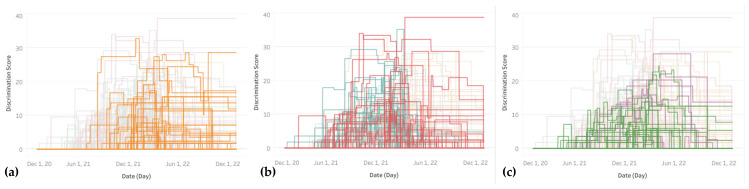
Timelines of discrimination levels in 2021–2022 grouped by continents, including from left to right: (**a**) Africa (orange), (**b**) Asia (red) and Europe (blue), and (**c**) North (green) and South America (purple).

When filtering the interactive line chart by continent (Figure 5), it appears that most African countries waited until September 2021 or later, while many Asian and European countries had started discriminatory policies by July or August 2021. South America lagged North America by several months in introducing distinctions by vaccination status, and Oceania started these policies in earnest only by the end of 2021.

**Figure 6:**
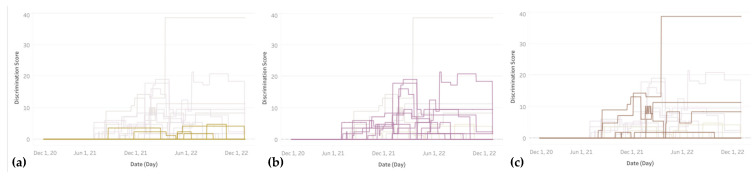
Timelines of discrimination levels in 2021–2022 grouped by sub-regions, including from left to right: (**a**) Eastern Asia, (**b**) South-Eastern Asia, and (**c**) Southern Asia.

We also replicated the 2021–2022 daily line chart with a filtering option by sub-region for more detailed geographic analysis. Figure 6 shows the discrimination timelines for three South- and East-Asian sub-regions. Eastern Asia, including Japan, South Korea, Hongkong, and Macao, is characterized by low levels of discrimination, which always remain below 5, about one half of the global average. This group also includes China and Mongolia with no discrimination throughout the entire time period. South-Eastern Asia presents a substantial degree of discrimination of unvaccinated citizens, with Indonesia, Laos, Malaysia, and the Philippines all reaching levels above 15 for at least one (Laos) to six (Philippines) months. Other Southeast-Asian countries stayed below levels of 10 (e.g., Singapore) or 5 (e.g., Cambodia) or avoided all differential restrictions (e.g., Thailand). Lastly, Southern Asia includes the top-10 country Pakistan with discrimination persisting at a very high level close to 40 for the better part of the year 2022. The other countries in this sub-region, including Bangladesh, India, Iran, Nepal, and Sri Lanka stayed below a moderate level of 15, with Afghanistan being an example of no recorded differentiation.

In addition to the analysis based on the CHI, we also examine the top-10 ranks, geographic patterns, and timelines of discrimination based on the OxCGRT Stringency Index. These patterns were very similar to those resulting from the CHI. The main difference were overall higher values based on the Stringency Index, as it contains a higher proportion of indicators where discriminatory policies were enacted.

## 4. Discussion

Based on an index calculated by subtracting the strength of containment and health policies for vaccinated people from those directed at the unvaccinated, a quantifiable degree of discrimination of unvaccinated citizens occurred in the majority of countries of the world during the COVID-19 pandemic. Discriminatory policies against unvaccinated citizens were enacted in more than 75% of national jurisdictions around the globe for at least a portion of the years 2021 and 2022. In contrast to an oft-repeated suggestion that many countries, particularly in Europe and North America acted in “lockstep”, the numeric, geographic, and temporal patterns of pandemic response policies tell a distinct story. Both the stringency and timing of the underlying policies and their variations targeting unvaccinated individuals greatly differed between countries. Generally, we observed greater stringency, as well as greater discrimination, across Europe and North America but also Asia and South America. The least amount of discrimination could be found in countries of Africa and Central America.

As discussed in the Introduction, the use of the term “discrimination” is not without limitations. Here, we refer to its literal meaning and to the lived experience of unvaccinated people during the pandemic. Furthermore, while rejecting coercive measures to get vaccinated against COVID-19, we acknowledge the inherent difficulty of balancing individual civil liberties with the perceived “common good” e.g., [13,61,62]. Due to the significant interference with bodily autonomy that an unwanted injection presents, we contend that the evidence for proportionate need, efficacy, and safety criteria of the COVID-19 vaccines was not demonstrated at any point at which unvaccinated people were excluded from services and social functions. Such criteria were established, for example, by the Public Health Agency of Canada [63] or the World Health Organization [64], which stated that it “does not presently support the direction of mandates for COVID-19 vaccination, having argued that it is better to work on information campaigns and making vaccines accessible”. (p. 1).

This research also has several technical limitations, as it is dependent on the quality and accuracy of the OxCGRT dataset, which we could not comprehensively verify. Assuming the source data provide a reasonable representation of each country’s policies, the definition of our index as the difference between the two existing index values could be refined, e.g., by differently weighting more and less consequential restrictions. Yet, this approach would require normative decisions on what services, venues, movements, and behaviours are more important than others and thus make the analysis more vulnerable to subjective bias than using the simple “area between the curves” approach based on the widely accepted OxCGRT index.

The definition of what constitutes “vaccinated” status may differ from one country to another. The data underlying our analysis represent each country’s policy stipulations with respect to the local definition of COVID-19 vaccination status. These definitions likely also changed over time, e.g., with the availability of booster shots. Note that OxCGRT recorded government policy, not voluntary measures such as businesses closures in the absence of a public health requirement to close. Furthermore, the OxCGRT project portrays policy enactment, not enforcement; thus, their data may not fully represent the situation on the ground.

## 5. Conclusions

In this exploratory study, we only looked at discrimination in terms of access to services and venues, as well as mobility restrictions for the general public. We did not include employment discrimination, which is a significant component of the discrimination experienced by unvaccinated citizens. For example, Chaufan and Hemsing [26] and Chaufan et al. [65] surveyed Canadian healthcare workers about the impact of workplace vaccination mandates on their employment, professional license, mental health, as well as patient care. To complement such work with quantitative analyses, we propose to study employment discrimination using the OxCGRT policy indicator V4, which recorded the requirement to be vaccinated in order to work in a specific occupation or for being a member of a specific population (e.g., age group or clinical risk group).

While this article focuses on the ethical and human rights issue of discriminatory pandemic response policies, further research could also examine the effectiveness of such policies. Some studies have measured positive effects of differential policies on vaccine uptake, e.g., [66]. We suggest investigating whether discriminatory public health interventions had any beneficial outcomes, by comparing our global index with an outcome measure such as excess mortality during the COVID-19 pandemic.

Based on our quantitative observation of non-negligible state discrimination against “the unvaccinated”, formal political and/or judicial investigations should be forthcoming as part of sustainable post-pandemic recovery. To prevent similar developments in the future, human rights protections need to be broadened to include all creeds, beliefs, political opinions, and lifestyles well beyond limited lists of “grounds” for the prohibition of discrimination that are prevalent in many current human rights codes. In turn, public health should re-focus on respect for individual autonomy and voluntary participation.

## Figures and Tables

**Table 1:** OxCGRT indicators included in the Containment and Health Index (CHI), based on Hale et al.’s [46] codebook and interpretation guidance.

Indicator	Differentiation Based on Vaccination Status	Coding Values	Flag Variable
C1 School closing (schools and universities)	Yes	0—no measures 1—recommend closing or open with alterations 2—require closing some 3—require closing all	0—targeted to specific region 1—across whole country
C2 Workplace closing	Yes	0—no measures 1—recommend closing or open with alterations 2—require closing for some 3—require closing for all—but—essential workplaces (e.g., grocery stores, doctors)	0—targeted to specific region 1—across whole country
C3 Cancel public events	Yes	0–no measures 1—recommend cancelling 2—require cancelling	0—targeted to specific region 1—across whole country
C4 Restrictions on gathering size	Yes	0—no restrictions 1—restrictions on very large gatherings > 1000 2—restrictions on gatherings > 100 3—restrictions on gatherings > 10 4—restrictions on gatherings of 10 people or less	0—targeted to specific region 1—across whole country
C5 Close public transport	Yes	0—no measures 1—recommend closing (or significantly reduce) 2—require closing	0—targeted to specific region 1—across whole country
C6 Stay-at-home requirements	Yes	0—no measures 1—recommend not leaving house 2—require not leaving house with exceptions for daily exercise, grocery shopping, and ’essential’ trips 3—require not leaving house with minimal exceptions (e.g., allowed to leave once a week, or only one person can leave at a time, etc.)	0—targeted to specific region 1—across whole country
C7 Restrictions on internal movement (between cities/regions)	Yes	0—no measures 1—recommend not to travel 2—restrictions in place	0—targeted to specific region 1—across whole country
C8 Restrictions on international travel (for foreign travellers, not citizens)	Yes	0—no restrictions 1—screening arrivals 2—quarantine arrivals from some or all regions 3—ban arrivals from some regions 4—ban on all regions	
H1 Public information campaign	No	0—no COVID-19 public information campaign 1—public officials urging caution 2—coordinated public information campaign	0—targeted to specific region 1—across whole country
H2 Testing policy (access to tests)	No	0—no testing policy 1—testing only those with symptoms AND specific criteria 2—testing of anyone with symptoms 3—open public testing	
H3 Contact tracing	No	0—no contact tracing 1—limited 2—comprehensive	
H6 Facial coverings	Yes	0—No policy 1—Recommended 2—Required in some shared/public spaces 3—Required in all shared/public spaces 4—Required outside the home at all times	0—targeted to specific region 1—across whole country
H7 Vaccination Policy	No	0—No availability 1—Availability for 1 of these 3 groups: key workers, clinically vulnerable, elderly 2—Availability for 2 of the groups 3—Availability for all 3 groups 4—Availability for all three groups plus more 5—Universal availability	0—At cost to individual (or funded by NGO, insurance, or partially government-funded) 1—No or minimal cost to individual (gov’t-funded or subsidized)
H8 Protection of elderly people	Yes	0—no measures 1—Recommended isolation, hygiene, and visitor restriction measures in LTCFs and/or elderly people to stay at home 2—Narrow restrictions for isolation, hygiene in LTCFs, some limitations on external visitors and/or restrictions protecting elderly people at home 3—Extensive restrictions for isolation and hygiene in LTCFs, all non-essential external visitors prohibited, and/or all elderly people required to stay at home and not leave the home with minimal exceptions, and receive no external visitors	0—targeted to specific region 1—across whole country

**Table 2:** The ten most discriminatory countries by 2021–2022 average and/or maximum value of the discrimination index calculated on the basis of the OxCGRT Containment and Health Index.

Rank (Top 10)	Average Discrimination Score		Maximum Discrimination Score	
1	Azerbaijan	18.70	Pakistan	38.69
2	Pakistan	18.22	France	35.12
3	Rwanda	12.37	Azerbaijan	33.93
4	Fiji	11.31	Saudi Arabia	33.34
5	Morocco	10.58	Cape Verde	32.73
6	Cape Verde	10.53	Rwanda	28.57
7	Saudi Arabia	10.40	Ecuador	27.98
8	Argentina	9.86	Turkey	27.97
9	Sierra Leone	9.52	Kosovo	27.38
10	Chile	9.28	Oman	26.78

## Data Availability

The original data are available from the sources cited in the Materials and Methods section. The authors’ version including the calculated discrimination index are posted to the Tableau Public visualization at https://public.tableau.com/app/profile/claus.rinner/viz/QuantifyingDiscrimination/Fig_2_Averagediscrimination (accessed on 15 March 2025). From there, the data including the discrimination index values can be downloaded by the user.
